# ﻿Adult morphology and redescription of *Lestidiopsindopacificus* (Ege, 1953), with comments on the features of related species (Teleostei, Aulopiformes, Paralepididae)

**DOI:** 10.3897/zookeys.1160.103110

**Published:** 2023-05-04

**Authors:** Hsuan-Ching Ho, Tzu-Yung Lin

**Affiliations:** 1 National Museum of Marine Biology and Aquarium, Pingtung, Taiwan National Kaohsiung University of Science and Technology Kaohsiung Taiwan; 2 Institute of Marine Biology, National Donghwa University, Pingtung, Taiwan National Museum of Marine Biology and Aquarium Pingtung Taiwan; 3 Australian Museum, Sydney, Australia National Donghwa University Pingtung Taiwan; 4 Department and Graduate Institute of Aquaculture, National Kaohsiung University of Science and Technology, Kaohsiung, Taiwan Australian Museum Sydney Australia

**Keywords:** Biodiversity, biogeography, ichthyofauna, ichthyology, taxonomy

## Abstract

Two specimens representing the first known adults of *Lestidiopsindopacificus* (Ege, 1953) are reported and described from Taiwan, and the validity and generic assignment of this species are confirmed. The origin of the pelvic fin directly below the dorsal-fin base shows that *L.indopacificus* belongs to the *L.mirabilis* species complex. It can be separated from its congeners by the position of the nostrils above the posterior end of the maxilla, the light body color with unevenly distributed melanophores in adults, and a distinct combination of meristic values and other morphological characteristics. New geographic records are reported for the two other current members of this species complex, *L.mirabilis* (Ege, 1933) and *L.extremus* (Ege, 1953). The diagnostic features that separate these three very similar species are discussed.

## ﻿Introduction

The barracudina genus *Lestidiops* was established by Hubbs (1916) but was assigned by Harry (1953a) to one of three subgenera in *Lestidium* before being elevated again by Rofen (1966) to a full genus. *Lestidiops* belongs to the naked paralepidids (subfamily Lestidiinae), which have a scaleless body except for the lateral-line, a single row of short teeth on the gill rakers, and more than 77 vertebrae. *Lestidiops* can be separated from the other genera of Lestidiinae by the following combination of features: head, snout, and body short to moderately slender; nostrils situated slightly to well before a vertical drawn through posterior end of maxilla; light organs and luminescent duct absent; anus close to tip of adpressed pelvic fin; anal-fin rays 25‒32; lateral-line usually incomplete (i.e. not reaching caudal-fin base, except in some species); well-developed ventral adipose fin present between anus and anal fin; and unevenly distributed melanophores to uniform black in adults (Harry 1953a; Rofen 1966; this study). There are about 25 nominal species of *Lestidiops*, but no conclusion has been reached as to how many are valid (Ho pers. data).

Recently, two adult specimens of barracudina collected from off southern Taiwan were observed to have the pelvic-fin origin below the dorsal-fin base and no light organs or luminescent duct. They were initially identified as *Lestidiopsmirabilis* (Ege, 1933) by the authors; however, both specimens had a much lighter body color compared to the uniformly dark brown to black body of specimens of *L.mirabilis*. More detailed examination has revealed that these two specimens represented the first adults specimens ever recorded of another, little-known species, *Lestidiopsindopacificus* (Ege, 1953).

*Lestidiopsindopacificus*, originally *Lestidiumindopacificum*, was described based on juveniles collected from the Indian Ocean (Ege 1953: fig. 30), with many larval specimens taken from widely scattered sites across the Indo-West Pacific. At 31.0 mm SL, the holotype has been the largest specimen available until now. Although many specimens in collections have been putatively identified as this species, it has not yet been possible to confirm these. Most literature records have been based on the original description, and others were likely misidentifications (Ho pers. data).

*Lestidiopsmirabilis*, originally *Paralepismirabilis*, was described by Ege (1933) solely on its holotype (whereabouts unknown), which had been collected from the Sulawesi Sea in the western Pacific. Ege (1953) provided more details of this species based on many juveniles (<47 mm SL) from the tropical Indo-Pacific and western Atlantic oceans. Adults of this species are rather rare, and only a few specimens are known from scattered localities (e.g., Harry 1953b; Uyeno et al. 1983; Gloerfelt-Tarp and Kailola 1984; Ho and Huang 2022). Some additional adult specimens from various localities were examined in the present study.

Recently, Ho and Huang (2022) provided the first description of an adult *Lestidiopsextremus* (Ege, 1953) from the Philippines and compared it with *L.mirabilis*. Another specimen from Myanmar, representing the first record of *L.extremus* in the eastern Indian Ocean, is reported in the present work.

This paper provides the first detailed description of adult-stage *L.indopacificus* and a comparison with its most closely related congeneric species. Morphological and biogeographical data pertaining to newly found specimens of *L.mirabilis* and *L.extremus* are also presented.

## ﻿Material and method

Counts and measurements were made following Ho and Golani (2019) and Ho et al. (2019a), with the addition of pre-nostril length measured from the tip of the snout to the center of the nostrils. The examined specimens are deposited in the Australian Museum, Sydney, Australia (**AMS**), Kochi University, Kochi, Japan (**BSKU**), the National Museum of Marine Biology and Aquarium, Pingtung, Taiwan (**NMMB-P**), the Department of Zoology, National Museum of Nature and Science, Tsukuba, Japan (**NSMT**), the South African Institute for Aquatic Biodiversity, Grahamstown, South Africa (**SAIAB**), and the Zoological Museum, Natural History Museum of Denmark, Copenhagen, Denmark (**ZMUC**).

Morphometric data were not taken from specimens in poor condition, including those that had been bent or damaged. Abbreviations: **DFO**, dorsal-fin origin; **AFO**, anal-fin origin; **VFO**, pelvic-fin origin. **D‒A**, horizontal distance between the origins of the dorsal and anal fins (= preanal length minus predorsal length) and **V‒A**, horizontal distance between the origins of the pelvic and anal fins (= preanal length minus prepelvic length). **PVLL**, **PDLL**, and **PALL** are numbers of lateral-line scales before VFO, DFO, and AFO, respectively, **TLL** is the total number of lateral-line scales, including large and small scales. **PVV**, **PDV**, and **PAV** are numbers of vertebrae before VFO, DFO, and AFO, respectively, and **PHV**, **CV**, and **TV** are numbers of prehaemal, caudal, and total vertebrae, respectively.

Data used for comparison are taken from Ege (1953), Harry (1953b), and Ho and Huang (2022).

## ﻿Results

### ﻿Family Paralepididae

**Identity as *Lestidiopsindopacificus***

The holotype of *Lestidiumindopacificum* (ZMUC P2329237) is in rather poor condition, with its dorsal fin missing entirely. Ventral adipose fins are present on the abdominal ridge and well developed between the anus and the AFO. The eyes are large, the snout is short, and the eye diameter is about half the snout length. There are 32 prepelvic vertebrae, 49 preanal vertebrae, and 80 total vertebrae counted directly from the translucent specimen. There is neither a light organ around the eye nor a luminescent duct inside the abdominal cavity. The anus is located close to the tips of the adpressed pelvic fins.

The original data for the holotype (31.0 mm SL) provided by Ege (1953) recorded the predorsal length as 53.9% SL and the prepelvic length as 54.8% SL, which implies that the VFO is slightly behind the DFO and under the dorsal-fin base. No drawing of the holotype was provided, but it is notable that the drawing of the largest specimen (21.1 mm SL) provided in Ege (1953: fig. 26) shows its VFO situated on about the same vertical line as its DFO, which is slightly different from the relative positions of the fin origins implied by Ege (1953) for the holotype.

Rofen (1966) later assigned the species to *Lestidiops*, a conclusion accepted here. The total of 80 vertebrae excludes it from Paralepidinae. The position of the VFO slightly behind the DFO excludes it from *Stemonosudis* and *Dolichosudis*. The presence of ventral adipose fins and the position of the anus close to the tips of the adpressed pelvic fins exclude it from *Macroparalepis*, and the lack of light organs or a luminescent duct excludes it from *Lestidium* and *Lestrolepis*. The position of the nostrils above or before the posterior end of the maxilla and the presence of a small pelvic fin exclude it from *Uncisudis*.

If the inferred position of the VFO in the holotype is accepted as standard, *L.indopacificus* shares this feature only with *L.mirabilis* and, by this alone, these two species can be separated from all other nominal congeners. According to Ege (1953), *L.indopacificus* has 79‒83 total vertebrae, 34‒36 prehaemal vertebrae, and 29‒32 anal-fin rays, data that match our two adult specimens well and which are clearly different from those of the two other current members of the *L.mirabilis* complex. In sum, all the available information allows the present specimens to be recognized as adults of *L.indopacificus*.

#### ﻿The *Lestidiopsmirabilis* complex

The three species reported below share a distinctive characteristic, namely, the VFO lies clearly behind the DFO, either below the dorsal-fin base or slightly behind that point. Rofen (1966) included *L.mirabilis* and *L.extremus* in the *extremus* complex (= *L.mirabilis* complex herein). However, many members of the scaled genera in the Paralepidinae have in common the VFO behind the DFO and the VFO usually under or slightly behind the dorsal-fin base (Post 1987; Ho and Duhamel 2019). Members of *Lestidiumatlanticum* complex, i.e. *L.atlanticum* Borodin, 1928, *L.orientale* Ho, Tsai & Li, 2019, and *L.longilucifer* Ho, Graham & Russell, 2020, usually have the VFO at about same vertical as the DFO, or with the VFO sometimes slightly before or behind the latter (Ho et al. 2020).

Based on our examination, the three current members of the *L.mirabilis* complex can be separated from other species of Paralepidinae by having 80‒87 vertebrae in totalal, a single row of small teeth on the gill rakers (vs multiple rows of teeth, slender in many species), and a naked body except for the lateral-line scales (vs a body fully covered with scales). They can be separated from members of the *L.atlanticum* complex by the lack of a luminescent duct inside the abdominal cavity (vs a long duct extending to the gular region).

All other members of *Lestidiops*, regardless of their taxonomic validity, have the VFO situated slightly or well before the DFO.

##### ﻿ Lestidiops indopacificus

Taxon classificationAnimaliaAulopiformesParalepididae

(Ege, 1953)

598D0AE0-9898-51DE-94B3-A9D367F425D3

**Figure 1.**
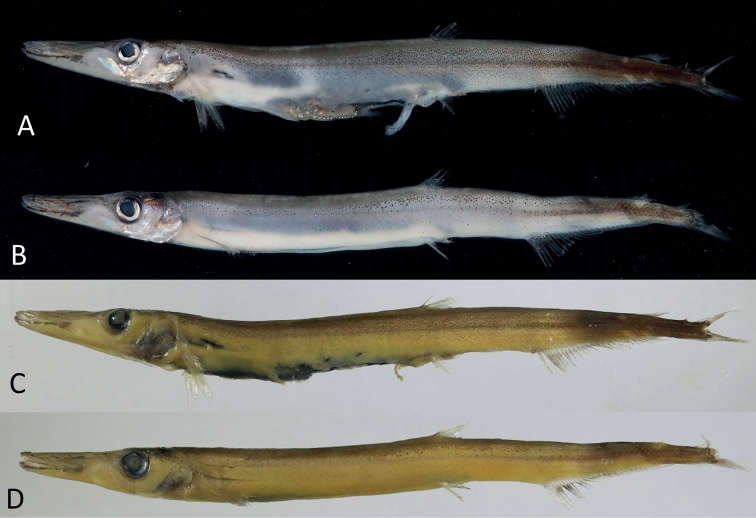
Adults of *Lestidiopsindopacificus* (Ege, 1953), NMMB-P34421 **A, C** 195 mm SL **B, D** 173 mm SL **A, B** fresh, photo by C.-N. Teng **C, D** preserved.

**Figure 2.**
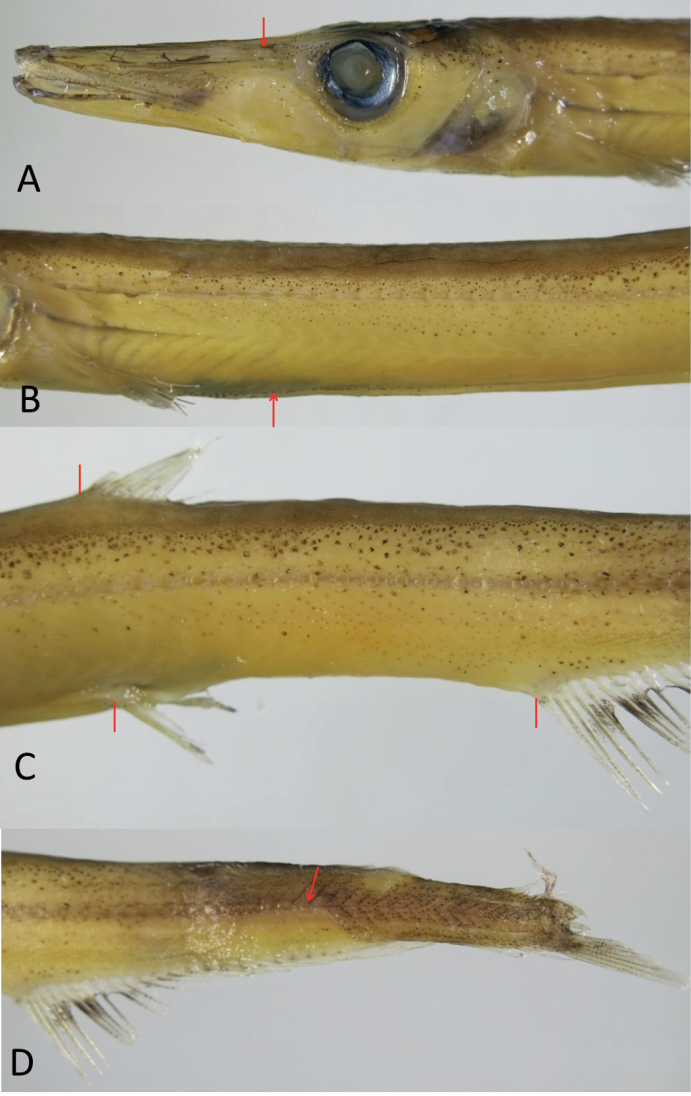
Close-up photos of *Lestidiopsindopacificus* (NMMB-P34421, 173 mm SL), left side with anterior to left, showing pigmentation and positional details of various structures **A** head, with arrow pointing to the nostrils **B** anterior trunk, arrow point to abdominal ridge **C** posterior trunk, with bars pointing to DFO (upper), VFO (lower left), and AFO (lower right) **D** tail, with arrow pointing to end of lateral line.

Figs 1 , 2 ; Tables 1 , 2 English name: Gray lightless barracudina

Lestidium indopacificum Ege, 1953: 120, fig. 26 (type locality: off India, Indian Ocean, 1°45'N, 73°03'E, ca 100 m depth).
Lestidiops indopacificus (Ege, 1953): Post 1972: 148 (type catalog).
Lestidiops indopacificum (Ege, 1953): Rofen 1966: 301 (presumably in Lestidiops, maybe related to L.mirabilis).
Lestidiops indopacificus (Ege, 1953): Paxton et al. 1989: 246 (Australia, probably misidentification); Nakabo 2000: 368 (Ryukyus, juveniles only); Fukui and Ozawa 2004: 293 (list); Mundy 2005: 203 (larvae); Gomon 2008: 267 (Australia, misidentification of other Lestidiops).

###### Material examined.

***Holotype***: ZMUC P2329237, in poor condition, 31.0 mm SL in original description. Adult specimens: NMMB-P34421 (2, 173‒195 mm SL), off Dong-gang, Pingtung, southwestern Taiwan, ca 300 m depth, 30 Jul. 2020.

###### Diagnosis.

Species of *Lestidiops* in the *L.mirabilis* species complex with the VFO under dorsal-fin base and anus farther posteriorly; dorsal portion of body and lateral line covered with melanophores, but large unpigmented areas present on lower portions of head and abdomen; lateral-line scales: PDLL 32‒33, PVLL33‒34, PALL 47‒48, TLL 66‒70; vertebrae: PDV 32‒33, PVV 33‒34, PAV 47‒48, PHV 34‒35, CV 44‒45, TV 80; total number of gill rakers 42‒44 (based on adult specimens). Juveniles without dark blotches or bands.

###### Description.

Morphometric and meristic data as provided in Tables 1 and 2. Dorsal-fin rays 10; pectoral-fin rays 13‒14; pelvic-fin rays 10; anal-fin rays 30. Lateral-line scales: PDLL 32‒33, PVLL 33‒34; PALL 47‒48; 66‒70 in total, with 58‒61 large scales and (in rear portion) 6‒9 small scales. Vertebrae: PDV 32‒33, PVV 33‒34, PAV 47‒48, PHV 34‒35, CV 44‒45, 80 in total. Gill rakers: 42‒44, with 8‒11 on upper limb (epibranchial) and 33‒34 on lower limb, including 21 on ceratobranchial + 12‒13 on hypobranchial.

**Table 1.** Morphometric data, as in % SL and % HL, of three species in the *L.mirabilis* complex. * including four specimens from Harry (1953b).

	*L.indopacificus*		*L.extremus*	*L.mirabilis*	
	NMMB-P34421		AMS I.36462-006	*n* = 8*	
SL	195	173	190	131.5‒276	SD
In % SL					
Head length (HL)	24.7	24.3	21.4	24.3 (22.7‒25.6)	0.9
Body depth	7.9	7.6	6.8	7.5 (7.1‒8.0)	0.4
Predorsal length	60.1	59.8	57.9	61.7 (59.9‒63.4)	1.1
Prepelvic length	60.8	60.7	63.2	61.9 (59.8‒63.6)	1.4
Preanal length	75.8	75.7	76.1	78.3 (75.9‒80.1)	1.2
V‒A	15.0	15.0	12.9	16.4 (15.2‒18.5)	1.1
D‒A	15.8	16.0	18.2	16.6 (14.5‒17.7)	1.1
Eye diameter	4.0	4.0	3.6	3.4 (3.1‒4.0)	0.3
Interorbital width	2.2	2.2	2.6	2.8 (2.5‒3.0)	0.2
Snout length	14.0	14.1	10.7	13.0 (12.3‒14.4)	0.7
Head depth	6.6	6.4	6.2	6.3 (5.6‒7.0)	0.6
Head width	3.4	3.5	–	3.8 (3.6‒4.1)	0.2
Upper jaw	12.1	12.1	10.4	12.4 (11.8‒13.1)	0.4
Lower jaw	16.2	16.9	13.6	16.2 (15.1‒17.3)	1.0
Pectoral-fin length	–	–	9.5	10.3 (8.3‒11.8)	1.1
Anal-fin base	17.5	17.7	17.9	15.5 (14.7‒16.3)	0.6
Dorsal-fin base	3.5	3.3	3.9	3.7 (3.4‒4.1)	0.3
Pre-nostril length	11.6	11.6	8.1	10.4 (10.2‒10.6)	0.3
Caudal peduncle depth	2.3	2.4	–	2.6 (2.4‒2.8)	0.2
Caudal peduncle length	6.1	6.6	5.6	4.3 (3.5‒5.2)	1.2
In % HL					
Pectoral-fin length	–	–	44.2	42.2 (34.3‒47.1)	4.6
Eye diameter	16.2	16.6	17.0	14.0 (13.0‒15.6)	0.9
Interorbital width	8.9	9.0	12.3	11.2 (9.9‒12.7)	1.0
Snout length	56.6	58.0	50.1	53.1 (49.0‒56.2)	2.4
Head depth	26.8	26.1	28.7	26.4 (25.1‒27.6)	1.2
Pre-nostril length	47.1	47.5	37.8	41.5	–
Upper jaw	49.0	49.6	48.4	50.7 (49.4‒52.2)	1.1
Lower jaw	65.4	69.4	63.6	66.3 (63.2‒67.9)	2.7

**Table 2.** Meristic data of three species of the *Lestidiopsmirabilis* species complex. Counts of both sides provided when different. * including four specimens from Harry (1953b). GR = gill rakers.

	*L.indopacificus*		*L.extremus*		*L.mirabilis*
	NMMB-P34421		AMS I.36462-006	SAIAB 203471	*n* = 9*
Dorsal-fin rays	10	10	10	10	9‒10
Anal-fin rays	30	30	31	30	28‒30
Pectoral-fin rays	14	13	12	13	12‒14
Pelvic-fin rays	10	10	10	10	9‒10
PVLL	33	33/34	38	37	35‒40
PDLL	32	32/33	34	32	36‒39
PALL	47	48	51	50	51‒56
TLL (large)	58/60	59/61	63	63/62	63‒67
TLL (small)	9/6	7/9	8	5/7	8‒22
TLL	67/66	66/70	71	68/69	73‒85
PHV	34	35	41	41	36‒42
CV	44	45	39	40	43‒45
PVV	33	34	38	38	36‒39
PDV	32	33	33	32	35‒38
PAV	47	48	50	50	50‒55
TV	80	80	80	81	81‒87
GR, epibranchial	8	11	9	9	10‒15
GR, ceratobranchial	21	21	15	16	20‒26
GR, hypobranchial	13	12	10	9	12‒25
Total GR	42	44	34	34	44‒61

Body moderately long, strongly compressed, depth at pectoral fin 12.7‒13.2 times in SL. Caudal peduncle short, its length 1.5‒1.6 times eye diameter. Ventral adipose fin very weakly developed along abdominal ridge between pectoral and pelvic fins, better developed on margin between pelvic and anal fins. Anus situated above tip of appressed pelvic fin (smaller specimens with anus slightly before fin tip), distance from VFO to anus about 3.5‒4.0 times in V‒A.

Head moderately slender, long-triangular, its length 4.0‒4.1 in SL; snout slender and pointed anteriorly, its length 1.7‒1.8 in HL. Mouth terminal, moderately large, its gape extending to about 1.5 times eye diameter in front of eye. Lower jaw slightly upturned at tip, with small distal tab of fleshy tissue. Eye small, its diameter 6.0‒6.2 in HL. No light organ around eye. First suborbital bone slender, fifth and sixth bones expanded posteriorly, and seventh small. Interorbital space narrow, its width 11.1‒11.2 in HL; some straight ridges present on top of head and snout. Two nostrils located directly above posterior end of maxilla, latter extending to point about 2/3 eye diameter in front of eye. Numerous sensory canals on snout, check, operculum, and jaws; numerous sensory pores on dorsal surface of snout and lower surface of lower jaw.

Gill filaments present on all four gill arches. Fourth arch mostly connected to gill chamber wall by membranes. Pseudobranchs present, their anterior halves included in a deep pocket.

DFO slightly in front of VFO, predorsal length 1.7 in SL. Pectoral-fin base behind posterior margin of gill cover, upper end of fin base slightly below horizontal drawn through lower margin of eye; no small pocket behind fin base. VFO directly under dorsal-fin base, pre-pelvic fin length 1.6 in SL. No axial scale behind pelvic-fin base (probably lost during trawling). Anal fin originating in posterior fourth of body, pre-anal length 1.3 in SL. Dorsal adipose fin over rear portion of anal-fin base, its base length about equal to eye diameter.

Two or three small fangs at tip of upper jaw, followed by single row of numerous small, retrorse teeth, these gradually becoming smaller on posterior part of jaw. Vomerine teeth absent. One or two fangs (either depressible or fixed) at front end of each lower jaw, followed by two rows of fangs arranged in about 6 pairs (more teeth arranged irregularly on right jaw of larger specimen); those of inner row long with knife-like tip and depressible; those in outer row much shorter, curved, and fixed, slightly embedded in tissue. Two rows of fangs on each palatine with anteriormost teeth arranged in 4 widely spaced pairs, those in outer row long and depressible, those in outer row small and fixed; single row of small, widely spaced, fixed teeth on posterior part of palatine. One row of small, straight teeth on each side of tongue.

Shield-shaped gill rakers present on epibranchial, ceratobranchial, and hypobranchial parts of each gill arch, each raker with 3‒5 (rarely fewer) small teeth on broad base, these teeth barely extending upward beyond margin of gill arch. Teeth on pharyngeal arch short, arranged in oval patch with about 4 rows in middle. Single row of small teeth on fifth ceratobranchial.

Body scaleless, except for single row of lateral-line scales extending from above pectoral girdle to point above approximately two-thirds length of anal-fin base. Lateral-line scales slightly longer than wide, gradually becoming smaller and narrower posteriorly; 2 large pores on each upper and lower corner of most scales and 1 pore at front and 1 pore on each upper and lower corners of those scales on about posterior third of lateral line.

Luminescent duct absent.

###### Coloration.

When fresh, body pale to light gray, somewhat translucent, unevenly covered with melanophores (Fig. 1A, B), and with abdomen bright white. Dorsal fourth of body densely covered with tiny melanophores; slightly larger melanophores beneath extending to lateral line and further downward, gradually becoming scattered; lower third of abdomen pale with large unpigmented areas. Dense melanophores on dorsal surface of head, anterior snout and jaws, and gular region; rest of head pale, with scattered melanophores except in large unpigmented areas on cheek and operculum. Inner surface of operculum pale with large black patch; mouth cavity pale. Abdominal ridge covered with dense melanophores (fewer and scattered in small specimens). Pectoral, dorsal and pelvic fins pale, with scattered melanophores; anal fin covered with dense melanophores except for the paler basal region; caudal fin covered with dense melanophores. When preserved, body light yellow in general, with pigmentation pattern similar to fresh condition (Figs 1C, D, 2A‒D). Peritoneal membranes and stomach black; intestine pale.

###### Distribution.

Juveniles widespread in the Indo-West Pacific from Taiwan to Australia and South Africa to French Polynesia (Ege 1953). Adults known only from two specimens collected from off southwestern Taiwan by bottom trawl at an estimated depth of around 300 m based on other fishes collected in the bycatch.

##### ﻿ Lestidiops mirabilis

Taxon classificationAnimaliaAulopiformesParalepididae

(Ege, 1933)

1302314F-778E-52A7-AD16-442F5F2ECC47

**Figure 3.**
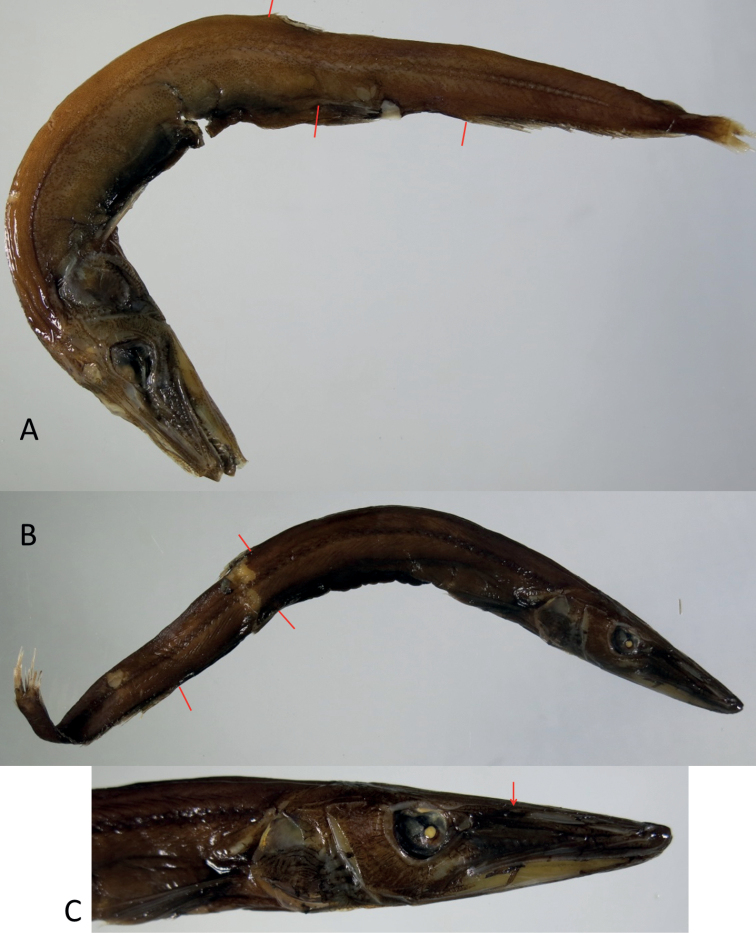
Photographs of two *Lestidiops* from Myanmar **A***L.extremus* (Ege, 1953), SAIAB 203471, ca 157 mm SL, with bars indicating DFO (upper), VFO (lower left), and AFO (lower right) **B***L.mirabilis* (Ege, 1933), SAIAB 203473, ca 180 mm SL, with bars indicating DFO (upper), VFO (lower right), and AFO (lower left) **C** head of specimen as in **B**, with arrow pointing to nostrils.

Fig. 3A ; Tables 1 , 2 English name: Strange pike smelt

Paralepis mirabilis Ege, 1933: 228 (type locality: Sulawesi Sea, western Pacific, 4°03'N, 123°26'E; holotype lost).
Lestidium mirabile (Ege, 1933): Harry 1953a: 240 (assigned to Lestidium). Harry 1953b: 197 (record from Hawaii, adults).
Lestidiops mirabilis (Ege, 1933): Rofen 1966: 331, 337 (assigned to Lestidiops). Post 1972: 142 (list; type lost). Uyeno et al. 1983: 193 (French Guiana and Suriname, adults). Gloerfelt-Tarp and Kailola 1984 (off Java, Indonesia, adult). Nakabo 2000: 368 (Kyushu, Japan, based on larvae). Ho and Huang 2022: 579 (Australia, adult).

###### Material examined.

***Non*-*types***: AMS I. 29537-004 (1, 210), FRV Kapala, 33°46'S, 151°49'E, east of Sydney, New South Wales, Australia, prawn trawl, 424‒439 m, depth 16 Dec. 1980 • BSKU 27461 (1, 238), 27°43'N, 126°26'E, Okinawa Trough, 1,100 m depth, 9 Mar. 1978. NSMT-P 40253 (1, 261), off Suriname, no other data (possibly reported by Uyeno et al. 1983) • SAIAB 82055 (1, 276), 23°57'36"S, 35°51'36"E, off Mozambique, Western Indian Ocean, 814‒831 m depth, 13 Oct. 2007. SAIAB 203473 (1, ca 180), 10°20'02.4"N, 96°24'14.4"E, Myanmar, Andaman Sea, 28 May 2015.

###### Other material

**(not examined but with reliable identification).** BMNH 1984.1.1.50 (1, 145), 8°45'S, 114°17'E, south of Java, Indonesia, eastern Indian Ocean (listed by Gloerfelt-Tarp and Kailola 1984) • USNM 163317 (1, 163), CAS-SU18635 (1, 131.5), ANSP 72175 (1, 170), and POFI 628 (1, 170), all from Hawaii (listed by Harry 1953b; Rofen 1966).

###### Diagnosis.

Species of *Lestidiops* with PVO below dorsal-fin base, anus slightly behind the base; anal-fin rays 28‒30; lateral-line scales: PVLL 35‒40, PDLL 36‒39, PALL 51‒56, TLL 63‒67+8‒22 = 73‒85; vertebrae: PHV 36‒42, PDV 35‒38, PVV 36‒39, PAV 50‒55, CV 43‒45, TV 81‒87; total gill rakers 44‒61. Adults with body uniformly dark brown to black, densely and evenly covered with melanophores; juveniles pale with broad, dark bands.

###### Distribution.

According to Ege (1953), circumglobal, including the western Atlantic, Indian, and central Pacific oceans based on larvae and juveniles, but more or less restricted to tropical regions. Adult specimens recorded from Indonesia (Gloerfelt-Tarp and Kailola 1984), Hawaii (Harry 1953b), Australia (Ho and Huang 2022), and Suriname and French Guiana (Uyeno et al. 1983); newly reported from Okinawa Trough, Myanmar, and Mozambique in present study. Bathymertic range 424‒1,100 m for adults.

###### Remarks.

The information on this species in Tables 1, 2 combines literature data with measurements and counts from examination of voucher specimens in earlier reports (e.g., Harry 1953b; Ho and Huang 2022) and additional specimens. Although *L.mirabilis* is a widespread species, adults appear to be rare, with only about 10 specimens known in collections. The specimen from Mozambique has the most lateral-line scales (85, including 63 large + 22 small), whereas others have 73‒80 (64‒67 + 8‒16). The number of large scales is fairly constant among specimens, but because most individuals were collected by bottom trawl and either suffered some degree of skin damage or had their body partly broken, it is possible that small scales at the posterior end of the lateral line are missing in some.

Harry (1953b) counted 11‒13 pectoral-fin rays in four adults, whereas 13‒14 such fin rays were counted in the present material. The first pectoral-fin element of paralepidids has two rays that are closely attached to each other, as can be seen in stained specimens (Ho et al. 2019a); Harry (1953b) probably counted them as a single ray, and, if so, his count should have been 12‒14 rays.

The number of gill rakers also shows a large variation. The specimen from Suriname has 44 gill rakers in total (12 on the upper limb + 32 on the lower), whereas other individuals have 53‒61 gill rakers (10‒14 + 39‒51). Rofen (1966) noticed that the vertebral counts are different among specimens from the western Atlantic Ocean (81‒83) and New Caledonia (86), which he concluded was due to lack of data, not to the existence of different geographic populations. Our only western Atlantic specimen also has 81 vertebrae, whereas three Pacific specimens have 86 or 87 vertebrae. Examination of more specimens may prove that there are consistent differences among populations.

The only specimen reported from New Zealand (Roberts et al. 2015) shows multiple rows of fine teeth forming tooth plates (in key) and is likely a misidentification of an *Arctozenus* species.

##### ﻿ Lestidiops extremus

Taxon classificationAnimaliaAulopiformesParalepididae

(Ege, 1953)

9F6B6C17-4AC7-573F-9252-D2370625131C

Fig. 3B ; Tables 1 , 2 English name: Extreme lightless barracudina

Lestidium extremum Ege, 1953: 136, fig. 29 (type locality: Molucca Passage, 0°18'S, 132°52'E [West Papua, Indonesia], ca 100 m depth). Rofen 1966: 301 (listed). Post 1972: 147 (type catalog; in Lestidiops, but possibly referable to Uncisudis).
Lestidiops extremus (Ege, 1953): Fukui and Ozawa 2004: 293 (list). Ho and Huang 2022: 580 (redescription, adult).

###### Material examined.

***Holotype***: ZMUC P.2136999 (21.8 mm TL), 0°18'S, 132°52'E, West Papua, Indonesia, ca 100 m.

***Non*-*types***: AMS I.36462-006 (1, 190 mm SL), about 80 km northwest off Camarines Norte Province, southeastern Luzon, Philippines, 14°50.46'N, 123°17.30'E‒14°47.35'N, 123°22.33'E, otter trawl, 760‒770 m, 27 Sep. 1995 • SAIAB 203471 (1, ca. 157), 12°42'59.4"N, 96°36'30"E, Myanmar, Andaman Sea, 25 May 2015.

###### Diagnosis.

Species of *Lestidiops* with VFO clearly behind DFO and slightly behind posterior end of dorsal-fin base; anus well behind DFO; posterior end of maxilla extending nearly to vertical drawn through anterior margin of eye; lateral-line scales: PVLL 37‒38, PDLL 32‒34, PALL 50‒51, TLL 68‒71, including 62‒63 large + 5‒8 small scales; vertebrae: PHV 41, CV 39‒40, PVV 38, PDV 32‒33, PAL 50, TV 80‒81; gill rakers 9+25 = 34; anal-fin rays 30‒31. Adults uniformly black; juveniles with dark blotches and broad bands.

###### Distribution.

Type series from off West Papua, Indonesia, ca 100 m depth; adults collected from off southeastern Luzon Island, Philippines, at 760‒770 m depth.

###### Remarks.

This species was originally described from a type series consisting of three larvae from Indonesia, and the only previously known adult was reported from the Philippines by Ho and Huang (2022). This is a very rare species, with only these four specimens and one additional adult from Myanmar known. This Myanmar specimen represents the first record in the eastern Indian Ocean and suggests that *L.extremus* may have a broader distribution range in the Indo-west Pacific region than has been suspected.

## ﻿Discussion

Ege (1953) included four species in his *Lestidiumindopacificum-mirabilis* group, including *L.indopacificum*, *L.atlanticum*, *L.mirabilis*, and *L.extremum*. He (Ege 1953: 139) distinguished these four nominal species by the proportions of the predorsal, prepelvic, and preanal (measured from tip of snout to anus) lengths, and by the length to the origin of the anal fin (= preanal length herein). He also showed, however, that three of them have similar counts of prehaemal vertebrae, total vertebrae, and anal-fin rays (Ege 1953: 140, no data available for *L.extremum*). The present examination of adults of all three species shows that these length proportions change with growth and become quite similar, even overlapping, as the more slender body shape of adults is attained. Apart from *Lestidiumatlanticum*, which remains in that genus, the other three species were later reassigned to *Lestidiops* and are currently in the *L.mirabilis* species complex (Rofen 1966; Post 1972; this study). The differences between the adults of these three species can now be presented and are based both on the present observations and data from the literature.

The snout is quite slender in *L.indopacificus*, moderate in *L.mirabilis*, and rather stout in *L.extremus*. The VFO is slightly but clearly behind the dorsal-fin base in *L.extremus*, whereas it is under the dorsal-fin base in *L.indopacificus* and *L.mirabilis* (Ho and Huang 2022; this study). In *L.extremus*, the posterior end of the maxilla extends nearly to a vertical drawn through the anterior margin of eye, whereas in the two other species, the maxilla only reaches a point well before the eye.

There are 10 dorsal-fin rays in all three species, except that Harry (1953a) counted nine in a specimen of *L.mirabilis*. There are 28 or 29 (29‒31 in Ege 1953) anal-fin rays in *L.mirabilis*, 30 in *L.indopacificus*, and 30 or 31 in *L.extremum*. Examination of more adult specimens may reveal overlapping or coincident ranges of counts for these features.

Total gill raker counts for *L.mirabilis* are 44 (Atlantic specimen) or 53‒61 (Indo-Pacific specimens) versus 34 total gill rakers in two adults of *L.extremus* and 42‒44 in two adults of *L.indopacificus*. The gill rakers are quite closely spaced in *L.mirabilis*, widely spaced in *L.extremus*, and intermediate in *L.indopacificus*. On each gill raker there are usually three or four closely spaced teeth, which are quite long in *L.extremus*, only moderately so in *L.mirabilis*, and short in *L.indopacificus*.

Comparison of the proportional measurements of all three species in the *L.mirabilis* species complex (Table 1) shows that the head is smaller in *L.extremus* compared to the other two species. In addition, the predorsal length is small, and the prepelvic length larger, in *L.extremus*. While the distance between VFO and AFO (V‒A) is clearly smaller in this species than in the other two, the distance between DFO and AFO (D‒A) is large. The snout and both jaws are also shorter in *L.extremus* than in the other two species, and the pre-nostril length is smallest in *L.extremus* and longest in *L.indopacificus*, as reflected by the different positions of the nostrils in these species.

As for meristic values (Table 2), *L.indopacificus* has some clearly low vertebral counts (PHV 34‒35, PVV 33‒34, and PAV 47‒48) compared to the other two species, and the latter two counts are consistent with the relatively low lateral-line scale counts (PVLL 33‒34, PALL 47‒48), which also readily separate *L.indopacificus* from the other two species. *Lestidiopsextremus* has the lowest caudal vertebra count (CV 39‒40), which clearly separates it from the other two species, and *L.mirabilis* has more PDV (35‒38) and PDLL (36‒39) than the other two species. Finally, *L.mirabilis* has a higher total count of lateral line scales (TLL 73‒85) versus *L.indopacificus* (66‒70) and *L.extremus* (68‒71).

It is notable that both adult specimens of *L.indopacificus* have their nostrils directly above the posterior end of the maxilla, which is different from the two other species (with nostrils situated well before the end of the maxilla), as well as from most other species of *Lestidiops*, although information on this is lacking for some species (Harry 1953a). The more posterior nostril position of *L.indopacificus* is reminiscent of *Stemonosudis*, which was defined by Harry (1953a) as having the laterally positioned nostrils distinctly behind the posterior ends of the maxillae but later revised by Rofen (1966) to “nostrils varying in position from before to slightly behind a vertical from posterior tip of maxilla.” Ho et al. (2019b) found that members of the *S.rothschildi* species complex also have their nostrils situated above or clearly before the posterior ends of the maxillae. More investigation is needed to fully delineate the taxonomic boundary between *Lestidiops* and *Stemonosudis*.

## Supplementary Material

XML Treatment for Lestidiops indopacificus

XML Treatment for Lestidiops mirabilis

XML Treatment for Lestidiops extremus
